# Weighted gene co-expression network analysis reveals specific modules and hub genes related to neuropathic pain in dorsal root ganglions

**DOI:** 10.1042/BSR20191511

**Published:** 2019-11-13

**Authors:** Nan Cheng, Zheng Zhang, Yue Guo, Zhuo-Lin Qiu, Jing-Yi Du, Zi-Qing Hei, Xiang Li

**Affiliations:** Department of Anesthesiology, The Third Affiliated Hospital, Sun Yat-Sen University, Guangzhou, China

**Keywords:** bioinformatics, Hub genes, Neuropathic pain, Peripheral nerve injury

## Abstract

Neuropathic pain is a common, debilitating clinical issue. Here, the weighted gene co-expression network analysis (WGCNA) was used to identify the specific modules and hub genes that are related to neuropathic pain. The microarray dataset of a neuropathic rat model induced by tibial nerve transection (TNT), including dorsal root ganglion (DRG) tissues from TNT model (*n*=7) and sham (*n*=8) rats, was downloaded from the ArrayExpress database (E-MTAB-2260). The co-expression network modules were identified by the WGCNA package. The protein–protein interaction (PPI) network was constructed, and the node with highest level of connectivity in the network were identified as the hub gene. A total of 1739 genes and seven modules were identified. The most significant module was the brown module, which contained 215 genes that were primarily associated with the biological process (BP) of the defense response and molecular function of calcium ion binding. Furthermore, C–C motif chemokine ligand 2 (Ccl2), Fos and tissue inhibitor of metalloproteinase 1 (Timp1) which were identified as the hub genes in the PPI network and two subnetworks separately. The *in vivo* studies validated that mRNA and protein levels of Ccl2, Fos and Timp1 were up-regulated in DRG and spinal cord tissues after TNT. The present study offers novel insights into the molecular mechanisms of neuropathic pain in the context of peripheral nerve injury.

## Introduction

Neuropathic pain is one of the most frequent forms of pathological pain, and is normally induced by somatosensory system injury or dysfunction [1]. Approximately 7–10% of the general population is exposed to this condition, and nearly 20% of the population who complain about chronic pain actually has neuropathic pain [2]. However, it is still largely unknown which critical genes are responsible for this condition and how the interactions among the critical genes induce the development of neuropathic pain. Therefore, a deep understanding of the molecular mechanisms of neuropathic pain is crucial for the development of an effective analgesic strategy.

Weighted gene co-expression network analysis (WGCNA) is a systematical method that was originally reported by Langfelder and Horvath [3]. This method is able to identify the correlation patterns of large and high-dimensional gene expression datasets [3]. WGCNA can group genes into a model or network according to the pairwise correlations among genes based on the similarities in the expression profiles. This modeling can be further correlated with clinical information, such as the stage of disease. In the last few years, WGCNA has been widely used to detect co-expressed modules and hub genes in various diseases including cancer [4], coronary artery disease [5] and peripheral arterial disease [6].

Dorsal root ganglions (DRGs) and spinal cord are considered to be key participants in the pathogenesis that lead to neuropathic pain [2]. Identifying the key genes and their interactions in the DRG and spinal cord might provide great insights into the underlying molecular mechanisms, which, in turn, could be crucial for the development of novel and efficient treatments for neuropathic pain. However, few studies have been performed using WGCNA to identify the significant modules and highly connected hub genes in DRG and spinal cord for neuropathic pain. In the present study, we applied WGCNA to construct a gene co-expression network and identify the significant modules based on a gene expression dataset of neuropathic pain from rat DRG and spinal cord. In addition, key genes and pathways in the most significant module were also identified.

## Materials and methods

### Microarray data sources and processing

The microarray dataset of neuropathic pain (E-MTAB-2260), published by Jamieson and colleagues [7], was downloaded from the ArrayExpress database (http://www.ebi.ac.uk/arrayexpress/), which includes 15 DRG tissues from tibial nerve transection (TNT) model (*n*=7) and sham (*n*=8) rats. All rats were confirmed to have tactile allodynia in response to mechanical pressure and the DRG tissues were harvested 7 days after surgery [7].

The downloaded raw dataset was analyzed with the Robust multi-array average (RMA) algorithm, using the software BRB-ArrayTools (version 4.5.1) [8]. To improve the robustness of the analysis, we excluded the following probes: (1) if less than 20% of the expression data had at least a two-fold change in either direction from the gene’s median value, or (2) the percentage of missing data or filtered out data exceeded 50%. Additionally, hierarchical clustering analysis was performed to assess the microarray data quality according to the distance between different samples in average linkage.

### WGCNA

We performed R package ‘WGCNA’ to construct weighted co-expression networks that could depict the correlation of gene expression patterns and highlight the highly correlated gene modules [3]. The detailed algorithm for the WGCNA construction was in accordance with the theory described by Zhang and Horvath [9]. Briefly, the Pearson’s correlation matrices were calculated between each gene pair. The pairwise correlation coefficient between the pair of genes m and n with the significance (Smn) was defined as Smn = |cor(m,n)|. Then, a power function (amn = power (Smn, β) = |Smn|β) was applied for the transformation of the Pearson’s correlation matrices to a weighted adjacency matrix. The parameter β was chosen based on the scale-free topology criterion. Subsequently, the adjacency matrix was transformed into a topological overlap matrix (TOM) with the appropriate β, and hierarchical average linkage clustering based on TOM was used to identify the gene co-expression modules that could group genes with similar expression profiles [10]. A heatmap plot of the corresponding eigengene network was drawn to analyze the relationships among the modules.

### Identification of the correlation between the modules and the pain condition

Two methods were employed to detect modules associated with the TNT condition. First, we considered the log_10_ transformation of the *P* value (lgP) in the linear regression between gene expression and clinical information as the gene significance (GS). Then the module significance (MS) was defined as the average GS of all genes in a module. The module with the highest absolute MS value among all the selected modules was considered to have significant association to the clinical trait (TNT condition) [4]. In addition, the module eigengenes (MEs) were considered to be the major components in the principal component analysis for each gene module and the expression patterns of all genes. The Pearson correlations between MEs and TNT condition were calculated to identify the module that was relevant to neuropathic pain. The *t* test was used to measure the significance of the Pearson correlation, and the modules with the *P*-values of less than 0.05 was indicated to have a significant correlation to TNT condition.

### Gene ontology and pathway enrichment analyses

Functional enrichment analysis for Gene Ontology (GO) and Kyoto Encyclopedia of Genes and Genomes (KEGG) pathways identification were performed through the R package clusterProfiler [11]. A Bonferroni corrected *P*-value of less than 0.05 was selected as the cut-off criterion for significant enrichment.

### Protein–protein interaction network analysis

We evaluated the relationship among genes in the significant modules from the perspective of protein interactions. The Search Tool for the Retrieval of Interacting Genes/Proteins (STRING; http://www.string-db.org) was used to identify the protein–protein interaction (PPI) network among these genes with a reliability threshold of more than 0.4 [12]. Subsequently, the PPI network was constructed and visualized using the Cytoscape software, and the subnetworks (modules) were identified using the Cytoscape plugin ClusterONE, with a threshold of less than 0.001. According to the method used in a previous study [13], we assessed the importance of a node in a PPI network and the subnetworks by its connectivity, i.e. the number of the proteins with which it was connected.

In addition, for each node in the PPI network, the gene expression intensities between TNT and sham samples were identified by the unpaired *t* test with the BRB-ArrayTools. The nominal significance level of 0.01 and absolute value of log_2_ FC (fold change) ≥1 were set as the thresholds.

### Rodent pain model

The protocol for rodent pain model was in accordance with the National Institutes of Health Guidelines for the Care and Use of Experimental Animals, and approved by the Animal Care Committee of Sun Yat-Sen University. All rats were provided by the Experimental Animal Center of Guangdong Province (Production license number: SCXK [Yue] 20180002), housed at 23 ± 2°C in separate cages with water and fed *ad libitum* in a 12-h reverse light cycle. We made every possible effort to minimize unnecessary suffering of animals.

The TNT surgery was performed on adult Sprague–Dawley rats (*n*=12) alongside sham controls (*n*=12) according to the previous study [14]. In short, from the exposed trifurcation of the left sciatic nerve, the tibial branch of the sciatic nerve was transected, whereas the sural and common peroneal nerves remained uninjured. The surgery for sham rats was consistent with TNT rats, except for the TNT. The rats were killed at 0 (before TNT or sham surgery), 3, 7 or 14 days after TNT surgery. The L5 DRG and lumbar enlargement (L4–L6) of the spinal cord tissues were harvested and snap-frozen with liquid nitrogen.

### Behavior test

As described by Hofmann and colleagues [14], measurement of the development and maintenance of paw withdrawal mechanical threshold (PWMT) was performed before TNT as basal responsiveness (day 0), and 1, 3, 5, 7, 10, and 14 days after TNT surgery by means of a pressure transducer (Electronic von Frey Anesthesiometer, IITC Company, New York, NY). The PWMT was measured once per trial and expressed as tolerance level in grams (g). During the test, the tip of the transducer was applied to the middle of the plantar surface of the operated hindpaw. Positive responses were defined as brisk withdrawal or paw flinching.

### Quantitative real‐time polymerase chain reaction

Quantitative real‐time polymerase chain reaction (qRT-PCR) was conducted as reported previously [15]. In short, total RNA was extracted from snap-frozen DRG and spinal cord tissues using TRIzol reagent (Thermo Fisher Scientific, Waltham, MA, U.S.A.). Reverse transcription was performed using ReverTra Ace qPCR RT Master Mix (Toyobo, Osaka, Japan). Quantitative analysis of target mRNA was conducted with qRT-PCR using SYBR® Green Realtime PCR Master Mix (Toyobo) with Roche LightCycler 1.1. The sense and antisense oligonucleotide primers for target mRNAs amplification were presented in Table 1. The housekeeping gene for qRT-PCR was Glyceraldehyde‐3‐phosphate dehydrogenase (*GAPDH*). All the samples were tested in quadruplicate, and differences between the target mRNAs and GAPDH were calculated with the 2^−ΔΔ*C*_t_^ method and normalized to the sham group at 0 day.

**Table 1:** The sequences of sense and antisense oligonucleotide primers

mRNA	Sense primer	Antisense primer
*Ccl2*	5′‐TGCTGCTACTCATTCACTGGC	5′‐CCTTATTGGGGTCAGCACAG
*Aif-1*	5′‐AGGCCACCAGCGTCTG	5′‐GCTGTACTTGGGATCATCGAG
*Timp1*	5′-CCCAACCCACCCACAGACAG	5′-GCCCGCGATGAGAAACTCCT
*GAPDH*	5′‐TCCTACCCCCAATGTATCCG	5′‐CCTTTAGTGGGCCCTCGG

Abbreviations: Aif-1, allograft inflammatory factor 1; Ccl2, C–C motif chemokine ligand 2; Timp1, tissue inhibior of metalloproteinase 1.

### Western blot analysis

Western blotting was performed following the standard procedures as previously described [15] by using the following antibodies: C–C motif chemokine ligand 2 (CCL2) (rabbit, 1:1000, Novus, Littleton, CO); allograft inflammatory factor 1 (AIF1; rabbit, 1:1000, Novus); tissue inhibitor of metalloproteinase 1 (TIMP1) (rabbit, 1:1000, Sigma–Aldrich, St. Louis, MO) and β-actin (1:3000; Sigma–Aldrich). All Western blots were repeated at least three times. Images were acquired by Tanon 5500 imaging system (Tanon, Shanghai, China). The images were scanned with the ImagePro Plus (version 6.0, National Institutes of Health, Bethesda, MD), the data are expressed as the values relative to the values of sham groups.

### Statistical analyses

The SPSS 20.0 software (SPSS Inc, Chicago, IL) was used for statistical analyses. Measurement data were expressed as mean ± standard deviation (SD). The PWMT data were analyzed statistically through the Mann–Whitney U test. The expressions of mRNA and protein among the groups were compared by one‐way analysis of variance (ANOVA) followed, where appropriate, by Tukey’s post hoc comparisons. Statistical significance was determined if *P*-value <0.05.

## Results

### Microarray data processing

Fifteen tissue sample files were downloaded from the ArrayExpress database and converted into expression data through the RMA algorithm, including background correction, quartile data normalization and summarization. Under the aforementioned filter threshold, a total of 1739 genes were obtained from the 15 samples for subsequent analysis.

### WGCNA and module identification

**Figure 1:**
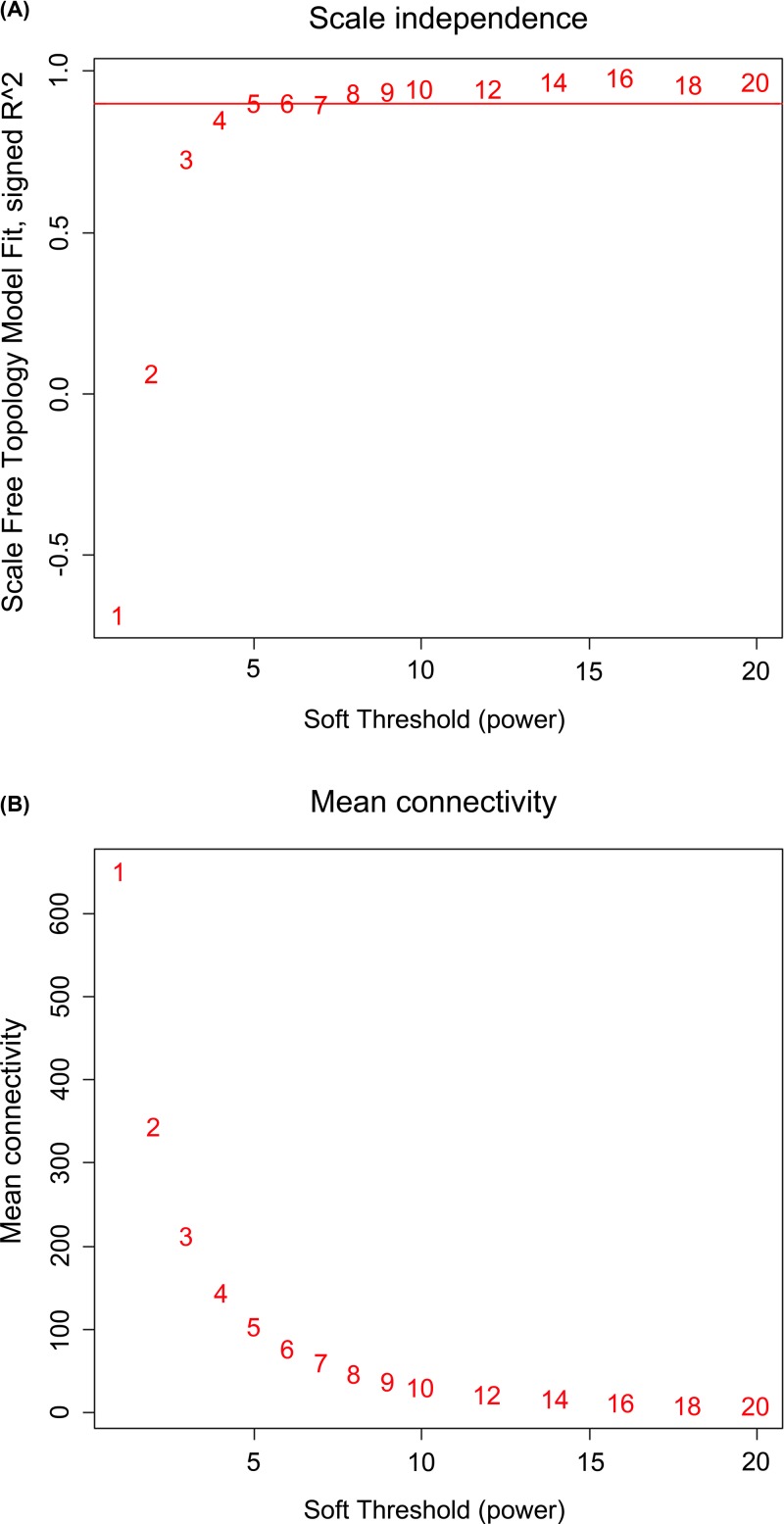
Network topology for different soft-thresholding powers The effect of different power values on the scale independence (**A**) and mean connectivity. (**B**) The approximate scale-free topology can be attained by using a soft-thresholding power of 5.

**Figure 2:**
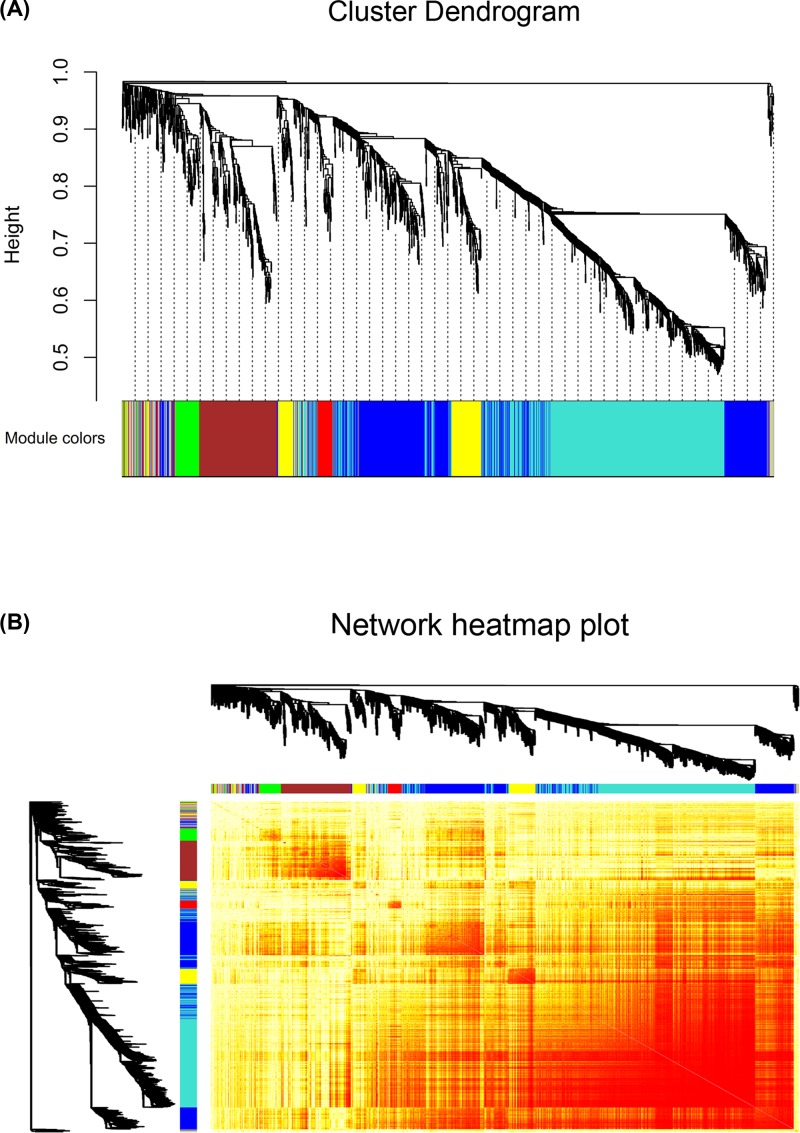
The co-expression modules and network heatmap plot constructed by WGCNA software (**A**) The gene dendrogram obtained by clustering the dissimilarity based on consensus topological overlapping with the corresponding module colors, indicated by the color row. Each branch in the figure represents one gene and every color below represents one co-expression module. A total of seven modules were identified. (**B**) Branches in the hierarchical clustering dendrograms correspond to each module. The brightness of yellow in the middle represents the connectivity degree of the different modules. Genes of high intramodular connectivity are located at the tip of the module branches because they show the highest interconnectedness with the rest of the genes in the module.

The soft threshold power was chosen to be five, based on the criterion of an approximate scale-free topology fit index 0.9 (Figure 1). The WGCNA package was used to construct co-expression modules. A total of seven modules were generated from the fifteen samples. These modules were labeled with colors and depicted in the dendrograms that are provided in Figure 2A. In addition, as shown in the network heatmap plot, each module exhibited independent validation to each other (Figure 2B).

**Figure 3:**
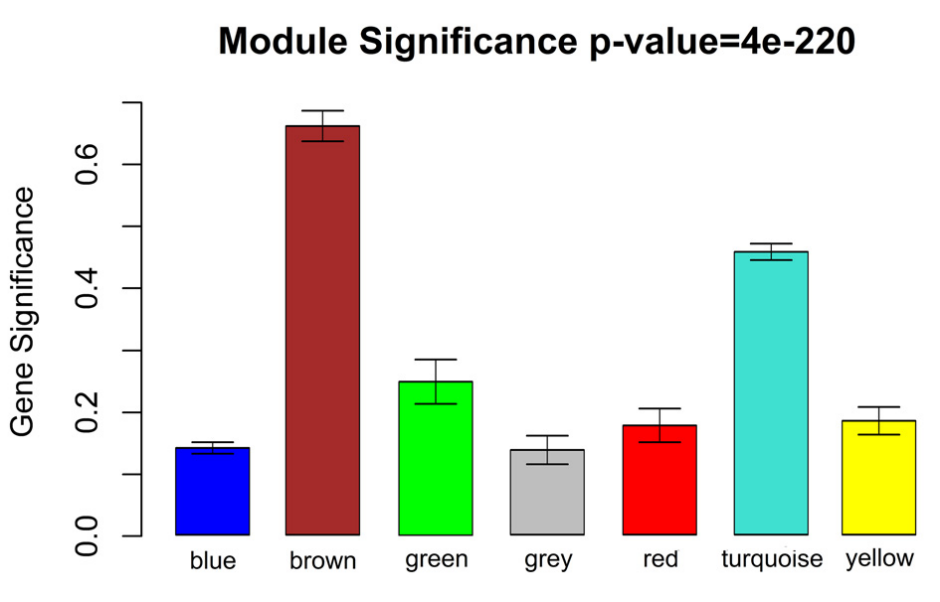
Identification of modules associated with neuropathic pain Distribution of average GS and errors in the modules associated with peripheral nerve injury. The horizontal axis indicates the module name and the vertical axis indicates the value of GS.

The correlation between each module and the TNT condition was tested by two methods, i.e. MS and heatmap of module–trait relationship. The MS of the brown module was the highest among all of the modules (Figure 3). Similarly, the genes clustered in the brown module were also found to exhibit the strongest positive correlation with the TNT condition (Table 2, r = 0.9, *P*=6.0e-6). Thus, it appears that the 215 genes in the brown module were significantly associated with the pain condition. Therefore, we mainly considered these genes in the following analysis for GO and pathway enrichment as well as PPI network.

**Table 2:** Correlations between modules and TNT

Module	Gene count	Correlation	*P*-value
Brown	215	0.9	6.0e-06
Green	72	0.33	0.2
Gray	72	0.097	0.7
Blue	500	0.029	0.9
Yellow	151	−0.067	0.8
Red	60	−0.19	0.5
Turquoise	669	−0.59	0.02

### GO and pathway enrichment analyses for the brown module

**Figure 4:**
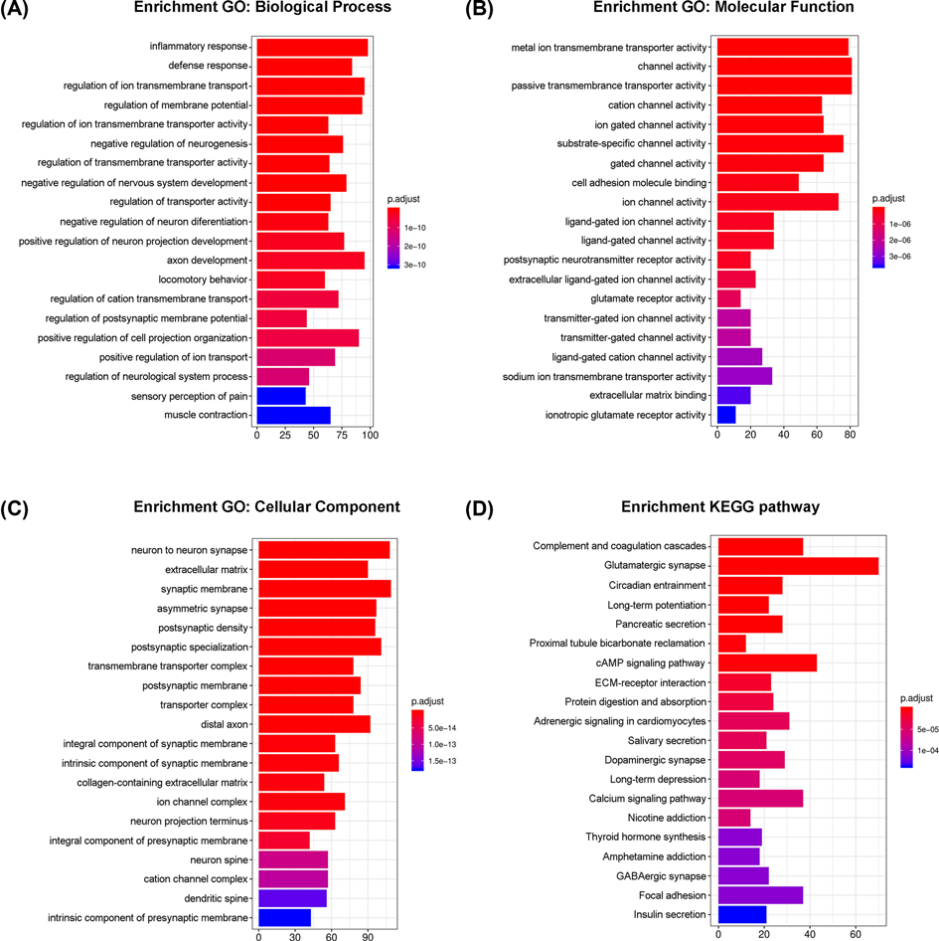
The most significantly enriched GO annotations and pathways of genes in the brown module The length of bars represents the numbers of genes, the color of bars corresponds to *P*-value according to legend. (**A**) The significantly enriched BP GO annotations. (**B**) The significantly enriched MF GO annotations. (**C**) The significantly enriched CC GO annotations. (**D**) The significantly enriched pathways.

Through the GO functional enrichment analysis (Figure 4), we found that regulation of inflammatory response (GO:0006954, *P*=2.957e-11), metal ion transmembrane transport activity (GO:0046873, *P*=4.229e-7) and neuron to neuron synapse (GO:0098984, *P*=2.144e-14) were the most significant enrichments in biological process (BP), molecular function (MF) and cellular component (CC) groups, respectively. In addition, these genes were also involved in the processes of ion transportion and regulations of channel activity, which have been proved to be related to neuropathic pain. The KEGG pathway enrichment analysis suggested complement and coagulation cascades (rno04610, *P*=8.124e-7) and glutamatergic synapse (rno04724, *P*=3.179e-6) were the top significant pathways. These results suggest that the genes in the brown module might be closely associated with the defense response and pain signal transportion among neurons after the peripheral nerve injury. Besides, the top significantly enriched GO terms and pathways for genes in each of the seven modules are shown in Table 3.

**Table 3:** The top significantly enriched GO terms and pathways for genes in each module

Module	GO						Pathway	
	Term (BP)	*P*-value	Term (MF)	*P*-value	Term (CC)	*P*-value	Term	*P*-value
Brown (gene count: 215)	Inflammatory response	2.957e-11	Metal ion transmembrane transport activity	4.229e-7	Neuron to neuron synapse	2.144e-14	Complement and coagulation cascades	8.124e-7
	Defense response	5.044e-11	Channel activity	5.719e-7	Extracellular matrix	4.743e-14	Glutamatergic synapse	3.179e-6
	Regulation of ion transmembrane transport	7.044e-11	Passive transmembrane transporter activity	6.397e-7	Synaptic membrane	4.743e-14	Circadian entrainment	2.777e-5
Green (gene count: 72)	No genes were enriched	No *P-*value	No genes were enriched	No *P-*value	No genes were enriched	No *P-*value	No genes were enriched	No *P-*value
Gray (gene count: 72)	No genes were enriched	No *P-*value	Receptor signaling complex scaffold activity	4.231e-3	Postsynaptic membrane	1.071e-3	No genes were enriched	No *P-*value
			Transmembrane receptor protein kinase activity	2.532e-3	Synaptic membrane	1.162e-3		
					Extracellular matrix	1.202e-3		
Blue (gene count: 500)	Regulation of transporter activity	1.077e-14	Drug transmembrane transporter activity	2.658e-5	Neuron to neuron synapse	1.886e-6	Glutamatergic synapse	2.971e-6
	Amino acid transport	1.421e-14	Postsynaptic neurotransmitter receptor activity	2.759e-5	Synaptic membrane	1.886e-6	Neuroactive ligand–receptor interaction	4.872e-5
	Neurotransmitter transport	2.254e-14	Structural constituent of postsynaptic specialization	5.778e-5	Postsynaptic specialization	2.965e-6	Circadian entrainment	4.871e-5
Yellow (gene count: 151)	Extracellular structure organization	5.787e-14	Extracellular matrix structural constituent	6.985e-9	Collagen-containing extracellular matrix	2.198e-18	ECM–receptor interaction	1.127e-7
	Extracellular matrix organization	1.778e-13	Platelet-derived growth factor binding	2.143e-6	Extracellular matrix	8.979e-18	Protein digestion and absorption	1.181e-7
	Collagen fibril organization	2.054e-7	Sphingolipid binding	1.211e-4	Collagen trimer	5.774e-12	Focal adhesion	8.861e-6
Red (gene count: 60)	No genes were enriched	No *P-*value	No genes were enriched	No *P-*value	*cis*-Golgi network	4.742e-2	No genes were enriched	No *P-*value
					Nuclear chromatin	4.742e-2		
Turquoise (gene count: 669)	Negative regulation of neuron differentiation	8.699e-8	Metal ion transmembrane transporter activity	1.974e-7	Neuron to neuron synapse	3.608e-13	Long-term depression	2.665e-4
	Multicellular organismal response to stress	2.247e-7	Cation channel activity	6.125e-7	Asymmetric synapse	1.604e-10	Proximal tubule bicarbonate reclamation	2.665e-4
	Negative regulation of nervous system development	4.403e-7	Substrate-specific channel activity	6.806e-6	Postsynaptic density	2.025e-10	Glutamatergic synapse	2.665e-4

### PPI network and subnetwork screening

**Figure 5:**
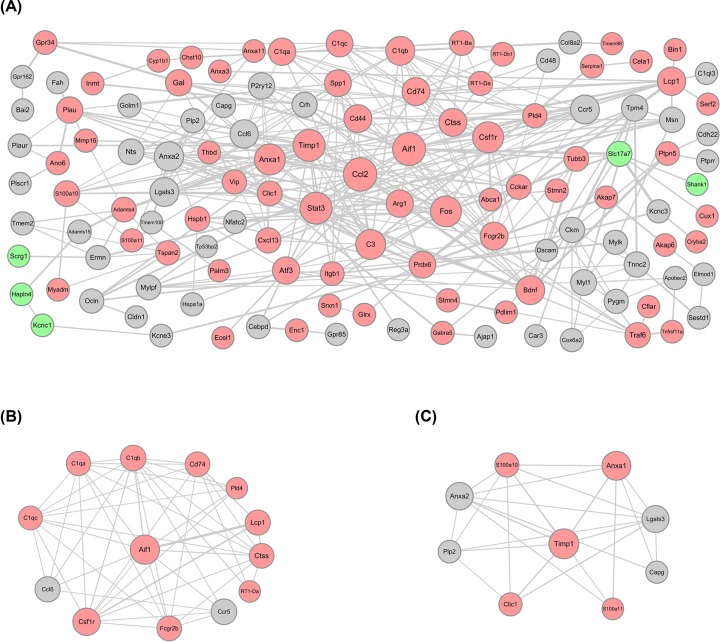
Protein modules in the PPI network The size of a protein is determined by the degree of its connection to other proteins, and the width of the edge connecting two proteins is determined by the combined score of the two proteins. The red circles represent up-regulated proteins, the green circles represent down-regulated proteins, and the gray circles represent the proteins without significant differential expression. (**A**) The PPI network constructed for genes in the brown module. (**B**) The subnetwork 1. (**C**) The subnetwork 2.

There were 125 nodes and 350 edges in the constructed PPI network for genes in the brown module (Figure 5A). The Ccl2 (degree = 23), Aif1 (degree = 22), Timp1 (degree = 20) and signal transducer and activator of transcription 3 (Stat3, degree = 20) were the top four proteins with relatively high connectivity. Furthermore, according to the subnetwork analysis by ClusterONE, two protein subnetworks were obtained (Figure 5B,C), namely subnetwork 1 (*P*=5.999e-5) and subnetwork 2 (*P*=3.937e-4). Aif1 and Timp1 were the crucial nodes with the highest connectivity in these two subnetworks, respectively. Therefore, we identified Ccl2, Aif1 and Timp1 as having the highest connectivity in the PPI network and two subnetworks separately as the hub genes for neuropathic pain in TNT.

### Behavioral tests

**Figure 6:**
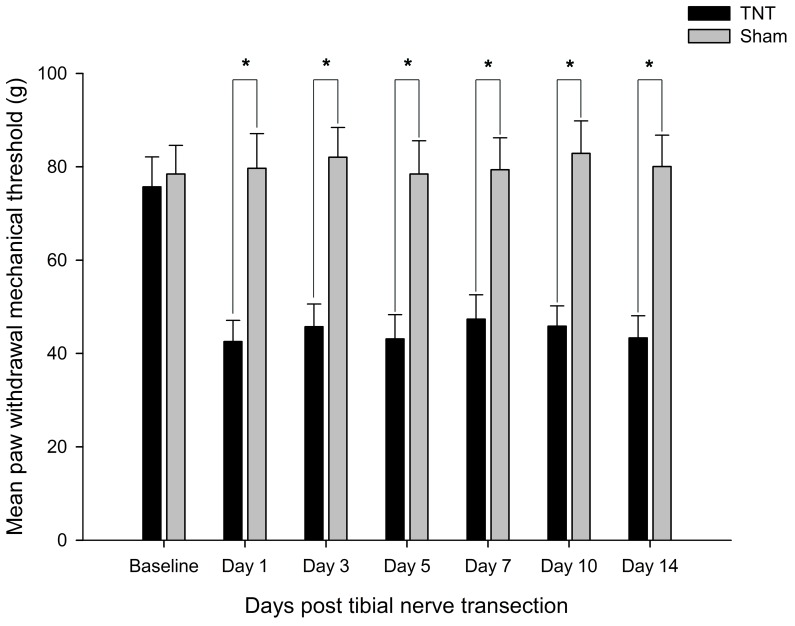
Time-course curves of mechanical allodynia induced by TNT TNT-induced significant decreases in PWMT in rats at 1, 3, 5, 7, 10, and 14 days after surgery. *P<0.05 vs the sham rats.

There was no significant differences in the comparisons of PWMT for both groups at baseline. Within 2 weeks post-surgery, the tibial nerve injury rats developed significant mechanical allodynia in the hind paw ipsilateral of injury at each time point, compared with the sham rats (Day 1, *P*=3.027e-5; Day 3, *P*=3.039e-5; Day 5, *P*=3.027e-5; Day 7, *P*=2.919e-5; Day 10, *P*=2.814e-5; Day 14, *P*=3.051e-5; Figure 6).

### Expression of hub genes in the DRGs after TNT

**Figure 7:**
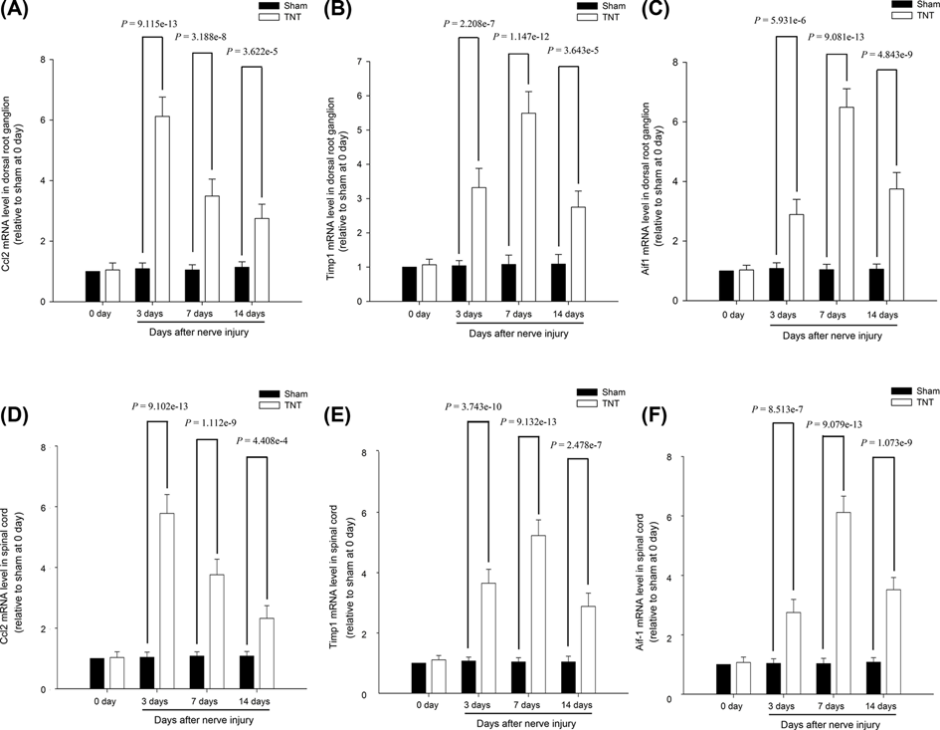
The mRNA expression levels for hub genes (**A**) Ccl2 mRNA expression level in the DRG. (**B**) Timp1 mRNA expression level in the DRG. (**C**) Aif1 mRNA expression level in the DRG. (**D**) Ccl2 mRNA expression level in the spinal cord. (**E**) Timp1 mRNA expression level in the spinal cord. (**F**) Aif1 mRNA expression level in the spinal cord.

**Figure 8:**
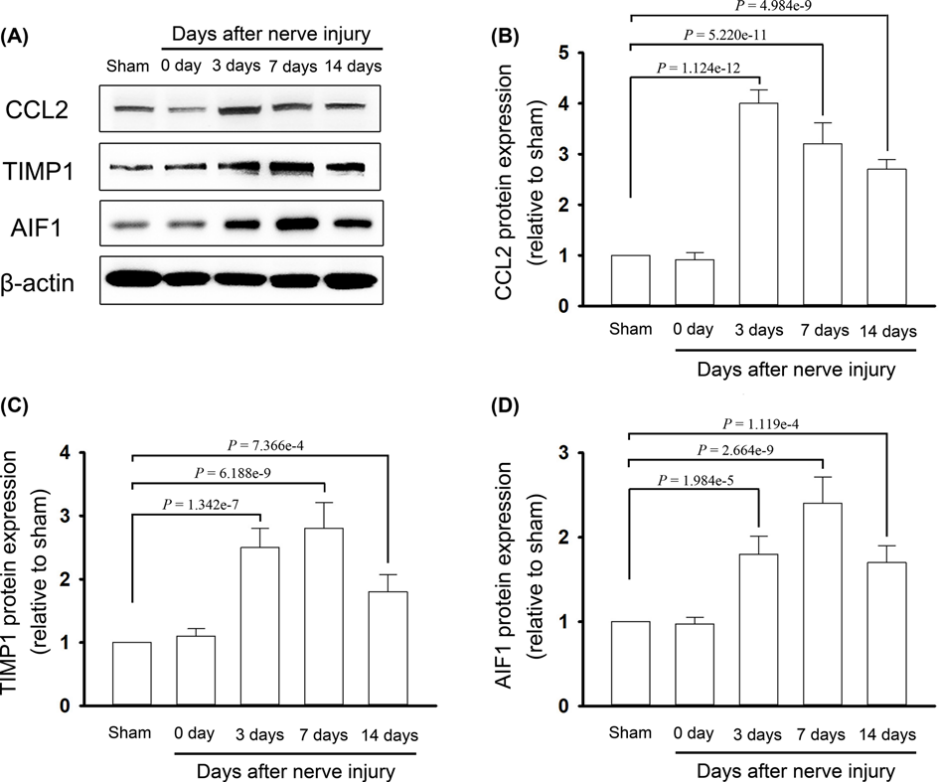
The protein expression levels for hub genes in the DRG (**A**) Protein expression for CCL2, TIMP1 and AIF1 was detected by the Western blot analysis. (**B**) Protein level of CCL2. (**C**) Protein level of TIMP1. (**D**) Protein level of AIF1.

**Figure 9:**
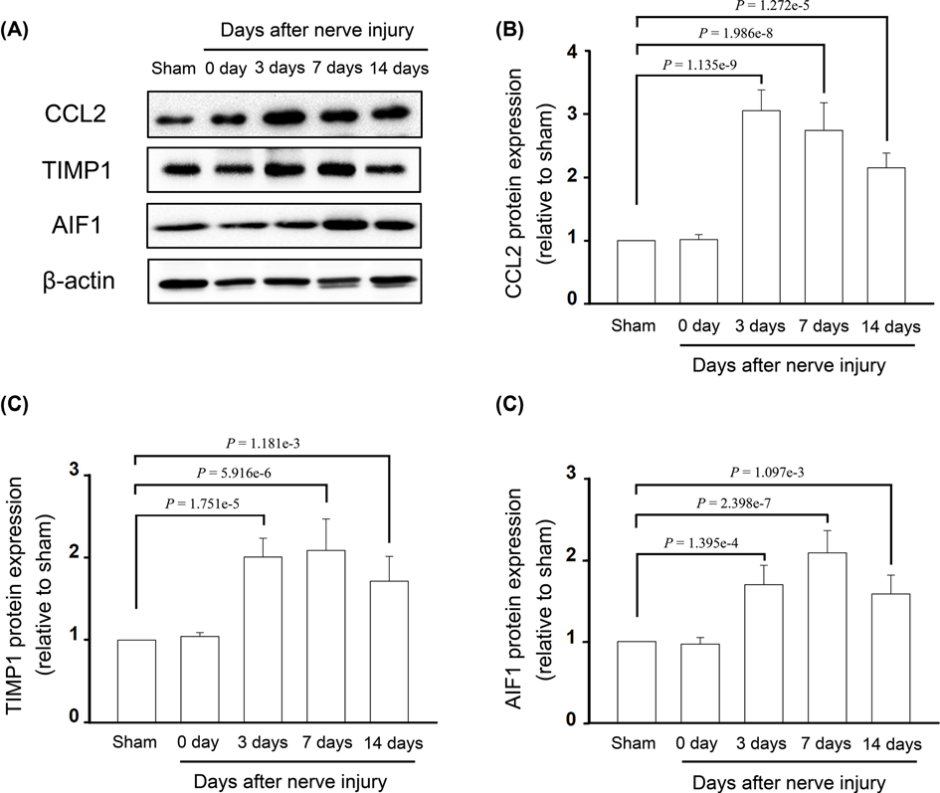
The protein expression levels for hub genes in the spinal cord (**A**) Protein expression for CCL2, TIMP1 and AIF1 was detected by the Western blot analysis. (**B**) Protein level of CCL2. (**C**) Protein level of TIMP1. (**D**) Protein level of AIF1.

As Ccl2, Aif1 and Timp1 were the nodes with highest connectivity degrees in the PPI network and two subnetworks, the expression of these three crucial nodes at both the mRNA and protein levels were, respectively, detected by qRT-PCR and Western blot analysis. At 3, 7 and 14 days after TNT, the expressions of Ccl2, Aif1 and Timp1 were significantly up-regulated in the DRG and spinal cord, compared with those in sham rats at the mRNA (Figure 7) and protein levels (Figures 8 and 9). The expressions of these three hub genes verified by qRT-PCR and Western blot analysis were identical with the bioinformatics analysis.

## Discussion

In the recent decades, gene expression profiling for neuropathic pain has been extensively documented. Furthermore, microarray technology combined with bioinformatics analysis allows for comprehensive analysis of the gene expression changes in neuropathic pain [13]. In the present study, co-expression patterns in neuropathic pain and the corresponding sham DRG as well as spinal cord tissues were identified using WGCNA, a powerful bioinformatics method. A total of seven co-expression modules were identified. Among them, the brown module was considered to be closely correlated with neuropathic pain in TNT injury. The genes in the brown module were further analyzed by GO annotation and pathway enrichment. In addition, three hub genes that identified through PPI network analysis and subnetwork screening were validated by qRT-PCR and Western blot.

Through the GO annotation enrichment analysis for 215 genes in the brown module, we found most of these genes were implicated in the defense and inflammatory response, which is in line with the results of a previous study that identified the association of genes in the defense and immune process in peripheral blood with pain in patients by WGCNA [16]. Furthermore, the activation of complement cascades is considered to play a vital role in the inflammatory and immune mechanisms of neuropathic pain. In fact, in this study, the most enriched KEGG pathway for genes in the brown module was the complement and coagulation cascades. This finding is in line with a previous study, which reported that most of the commonly regulated genes in neuropathic pain models that are induced by peripheral nerve injury are complement proteins, such as *C1q, C3* and *C4* [17].

Besides defense and inflammatory response, the genes in the brown module were also enriched in the process of ion activity regulation. It is well-known that metal ions such as calcium ion might regulate the function of the peripheral and central transmission of pain signals through the modulation of fast synaptic transmission and neuronal excitability [18,19]. In this study, we found that many genes in the brown module were crucial for ion transportion and channel activity. More specifically, some annexin family genes, such as annexin A2 and annexin A3, were found in the brown module and have been shown to mediate neuropathic pain through the regulation of transient receptor potential cation channel, subfamily A, member 1 (TRPA1)-dependent nociception and activation of microglia [20,21].

In addition, Ccl2, Aif1 and Timp1 were identified as the hub genes with highest connectivity in the PPI network and two subnetworks separately. Ccl2 is a chemokine that has been implicated in neuroinflammation and central sensitization through its preferred receptor C–C motif chemokine receptor 2 (Ccr2) [22]. In the neuropathic pain condition, Ccl2 can be transported from the DRG to a central terminal in the spinal cord, and Ccl2 released from primary afferents and astrocytes might be involve in microglial activation via Ccr2 in neurons leading to changes in the regulation of synaptic transmission [23,24].

Aif-1 is a novel inflammatory mediator that chiefly released by circulating monocytes and tissue macrophages during the inflammatory response processes under pathological conditions [25–27]. For nerve system diseases, such as spinal cord injury, the enhanced cytoplasmic AIF-1 immunoreactivity was demonstrated to activate microglial cells for the acute response to nerve system injury [28]. In the present study, the subnetwork revealed the connections of AIF-1 and some proteins such as Cathepsin S (CTSS) and C–C chemokine receptor type 5 (CCR5), and these proteins had been considered to play important roles in maintaining microglia activity in pain states [29,30]. Therefore, we hypothesized that the up-regulation of AIF-1 might be association with the microglia activation and proliferation in the neuropathic pain.

Timp1 is an inducible, soluble and secreted protein with cytokine-like properties. Previous study demonstrated that the expression of Timp1 significantly increases in the DRG of the rats after sciatic nerve transection (SNT), and that induced Timp1 mRNA is predominantly present in activating transcription factor 3 (ATF3)-positive injured DRG neurons [31]. When neuropathic pain occurred, the increasing of Timp1 expression might inhibit the expression of matrix metalloproteinases 9 (MMP-9), which promoted proliferation of the oligodendrocyte progenitors in the injured spinal cord and reverse allodynia [32]. Therefore, it seems that Timp1 is not only a biomarker, but a critical hub node in neuropathic pain.

## Conclusions

The current study used WGCNA to analyze the gene expression profile of in a rat neuropathic pain model induced by TNT, and identified seven co-expression modules. Among these modules, the brown module was closely associated with the neuropathic pain. Genes in the brown module are likely to be involved in the pathogenesis of neuropathic pain through the regulation of the defense response and calcium ion binding. Moreover, Ccl2, Aif-1 and Timp1 might be demonstrated as the hub genes with high connectivity. Future detailed functional studies are needed to confirm the contribution of these genes to neuropathic pain.

## Abbreviations

Aif1: allograft inflammatory factor 1
ATF3: activating transcription factor 3
Ccl2: C–C motif chemokine ligand 2
Ccr2/CCR5: C–C motif chemokine receptor 2/5
DRG: dorsal root ganglion
GAPDH: glyceraldehyde‐3‐phosphate dehydrogenase
GO: gene ontology
GS: gene significance
KEGG: Kyoto Encyclopedia of Genes and Genomes
ME: module eigengene
MS: module significance
PPI: protein–protein interaction
PWMT: paw withdrawal mechanical threshold
qRT-PCR: quantitative real‐time polymerase chain reaction
RMA: robust multi-array average
Timp1: tissue inhibitor of metalloproteinase 1
TNT: tibial nerve transection
TOM: topological overlap matrix
TRPA1: transient receptor potential cation channel, subfamily A, member 1
WGCNA: weighted gene co-expression network analysis
